# Supplementary material for: SARTools: A DESeq2- and EdgeR-Based R Pipeline for Comprehensive Differential Analysis of RNA-Seq Data
Source: PLoS One. 2016 Jun 9;11(6):e0157022. doi: 10.1371/journal.pone.0157022 (PMC4900645; doi:10.1371/journal.pone.0157022)
Supplement: S1 Appendix — This additional file shows why normalization methods are not interchangeable between statistical models without adequate transformation. (PDF) [file pone.0157022.s001.pdf]

Why are normalization methods not interchangeable?

Additional file 1 of *SARTools: a DESeq2- and edgeR-based R pipeline for comprehensive differential analysis of RNA-Seq data.*

1 Introduction

SARTools is a R package associated with two R script templates which allow one to perform differential analysis with either DESeq2 [1] or edgeR [2], the normalization method employed being the one associated with the package used. The purpose of this additional file is to show that normalization methods are not interchangeable between statistical models without adequate transformation. Despite this has been recalled in [3] some users of R packages for differential expression are still not aware of that. This is an important issue as DESeq(2) and edgeR use normalization factors in two different ways : DESeq(2) integrates the size factors in the calculation of the mean of the Negative Binomial distribution used to model raw counts when edgeR normalizes library sizes and includes them as an offset in the statistical model for differential testing. Therefore, normalization factors should be computed and used with regard to the statistical test that follows.

Here we compare the behaviour of two normalization methods (DESeq [4] and TMM [5]) associated with two different tests for differential analysis (DESeq2 and edgeR) with and without adequate transformation. For this comparison we used the four datasets from [6] (also available on GitHub: `https://github.com/PF2-pasteur-fr/SARToolsPaperData`). The properties and characteristics of these datasets are shown in table 1.

Organism	Type	Number of genes	Replicates per condition	Minimum library size	Maximum library size	Sequencing machine
H. sapiens	RNA	26 437	$\{3, 3\}$	2.0×10^7	2.8×10^7	Gallx
A. fumigatus	RNA	9 248	$\{2, 2\}$	8.6×10^6	2.9×10^7	HiSeq2000
E. histolytica	RNA	5 277	$\{3, 3\}$	2.1×10^7	3.3×10^7	HiSeq2000
M. musculus	miRNA	669	$\{3, 2, 2\}$	2.0×10^6	5.9×10^6	Gallx

Table 1: Short description of the data.

2 Comparison of normalized counts

For sake of simplicity we used the normalization terminologies proposed by DESeq2 and edgeR. DESeq2 uses *size factors* and applies them in the calculation of the mean of the Negative Binomial distribution used to model raw counts, when edgeR uses *normalization factors* to normalize *library sizes* (total number of reads) before integrating them as an offset in the statistical model. Thus, for each normalization method we computed a sizeFactor, i.e. a scaling coefficient that applies to raw counts, and a normalization factor, i.e. a scaling coefficient that applies to library sizes. We computed the normalization factors as follows with R:

TMM Trimmed Mean of M values. It has been derived for use with edgeR.

```
tmm <- calcNormFactors(geneCount, method="TMM")
```

RLE It is the DESeq normalization method adapted for use with edgeR.

```
rle <- calcNormFactors(geneCount, method="RLE")
```

and the size factors as follows:

DESeq DESeq normalization method as computed by DESeq(2).

```
dds <- estimateSizeFactors(dds)
deseq <- sizeFactors(dds)
```

TMM.counts TMM normalization factors transformed to be used with DESeq2.

`N <- colSums(geneCount)` (vector of library sizes)

```
tmm.counts <- N*tmm/exp(mean(log(N*tmm)))
```

In order to get normalized counts in the manner of DESeq2, these scaling coefficients were applied to raw counts by dividing each gene count by the sample-associated size factor or normalization factor:

$$x'_{ij} = \frac{x_{ij}}{\hat{s}_j} \tag{1}$$

where x_{ij} refers to the read count for gene i in sample j, and $\hat{s}_j$ is the size or normalization factor for sample j. This is equivalent to using the DESeq2 command `counts(dds, normalized=TRUE)` with specified size factors. As in [6] we used boxplots of normalized counts and the within group coefficient of variation to compare the distribution of the resulting normalized counts. Note that SARTools does not use these normalized counts as input but uses the raw read counts as constrained by DESeq2 and edgeR, and normalization is then performed with the package's dedicated function.

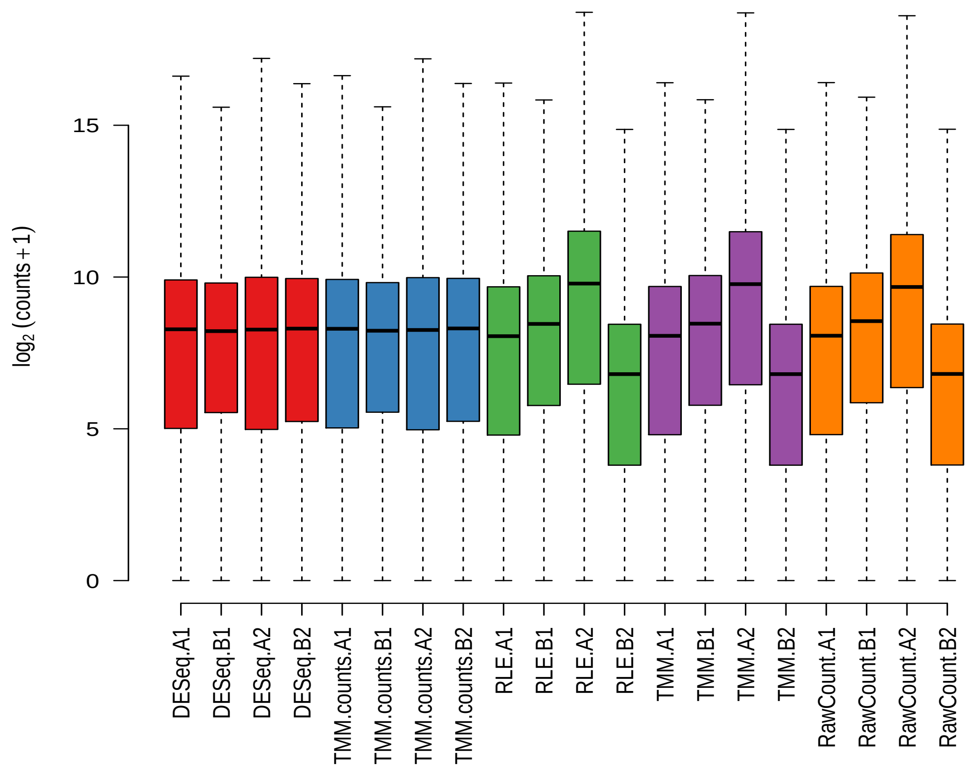

Figure 1: Boxplots of normalized counts for the Af dataset.

Figure 1 shows the results for the Af dataset. This dataset is composed of biological duplicates from two different biological conditions A and B. Scaling factors (DESeq and

TMM.counts) stabilize count distributions as opposed to an absence of normalization (rawCounts). In contrast and as expected, normalization factors do not perform well on counts as they are not adapted for such usage.

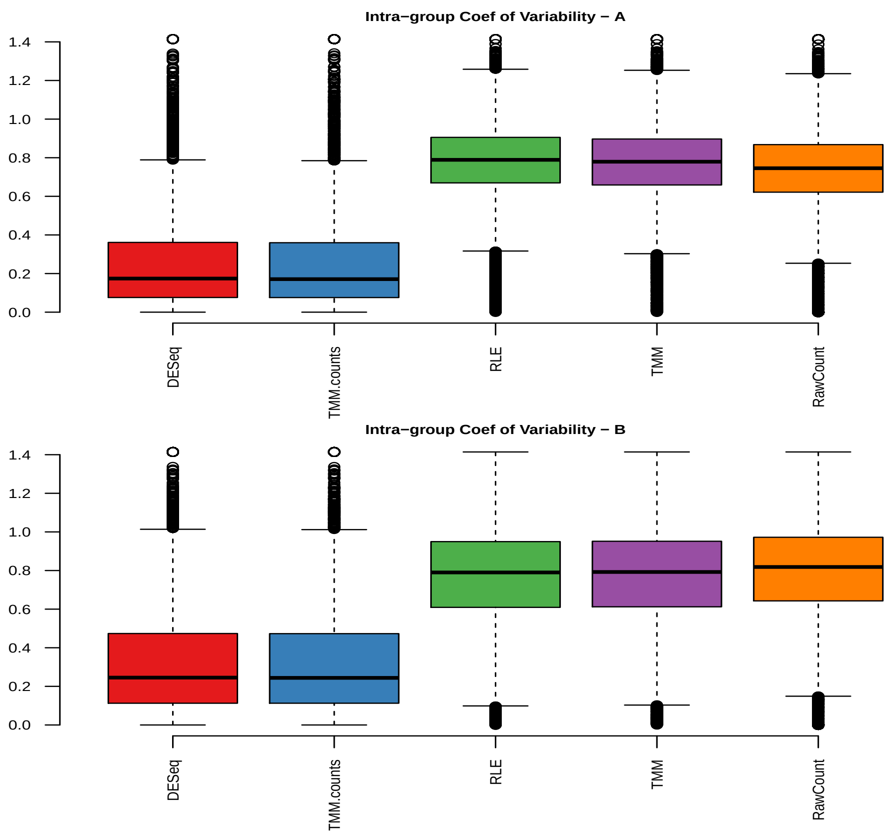

Figure 2: Boxplots of within-group coefficient of variability on the Af dataset.

Figure 2 (A and B) shows the distribution of the coefficient of variation across genes for each normalization method in each biological condition. Boxplots on this figure show a reduction of the within-group variability with respect to raw counts after a size factor-based normalization. This reduction is not observed after a normalization factor-based normalization. Both figures show that size factors and normalization factors do not perform similarly and must be properly transformed according to the statistical model used.

In this supplementary file, normalized read counts are extracted in order to be compared. This is not much-needed in a differential analysis, although this may be informative for biologists.

3 Comparison of lists of differentially expressed genes

The 4 normalization methods were used with two statistical tests for differential expression as follows:

DESeq2 they were used as scaling factors with:

```
sizeFactors(dds) <- scalingFactors
```

edgeR they were used as normalization factors with:

```
dge <- DGEList(geneCount, group=group, norm.factors=scalingFactors)
```

where `dds` and `dge` are the DESeq2 and edgeR data structures for count data respectively, `geneCount` is the matrix of raw counts, `group` is the vector of biological conditions for each sample and `scalingFactors` is either `tmm`, `rle`, `deseq` or `tmm.counts`.

We compared the resulting lists of DE genes ($\alpha = 5\%$) across the different analyses as explained in [6]. We computed the total number of DE genes for each combination of normalization method and differential test, and we computed the number of common genes between all pairs of methods. We then computed the Jaccard distance between each pair of lists of DE genes for each dataset and clustered the lists using a Ward criterion. We finally obtained a consensus clustering over the four datasets by averaging the Jaccard distance matrices to assess the similarity between the 8 methods (combination of the two statistical models and 4 normalizations).

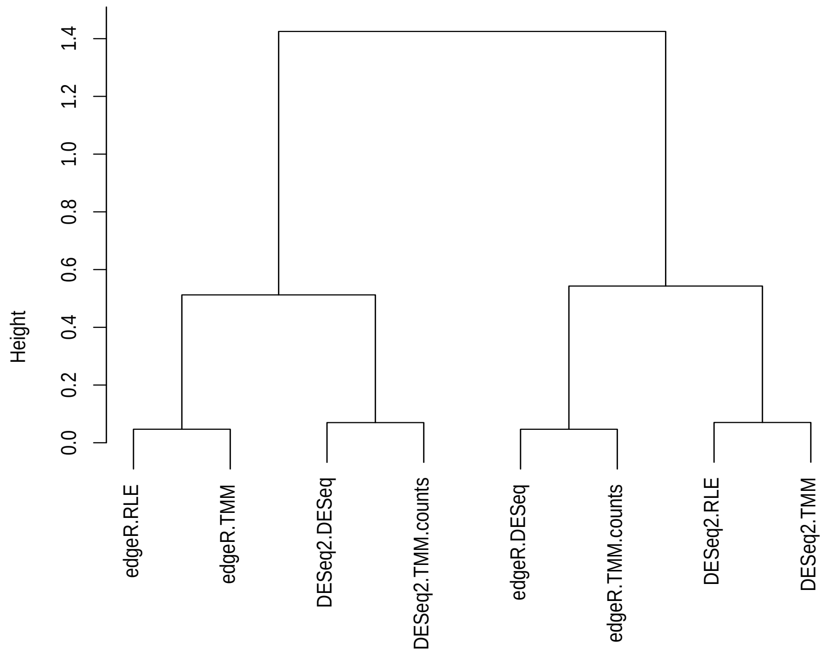

Figure 3: Consensus clustering of the 8 methods over the 4 datasets.

Table 2 shows the number of common genes between all pairs of methods for the Af dataset. As expected, size factors associated with DESeq2 provide similar results as normalization factors associated with edgeR. However we observe very important differences between these results and those obtained with the reversed combinations (size factors with edgeR and normalization factors with DESeq2). The same conclusion can be drawn from the consensus clustering (figure 3). This figure shows two distinct groups of combinations and demonstrates that these two groups are far more distant from each other than the normalization methods within each group. Therefore we conclude that it is of major importance to adapt the computation of this normalization coefficient when choosing a normalization method for a given RNA-Seq data set.

	DESeq2.DESeq	DESeq2.TMM.counts	DESeq2.RLE	DESeq2.TMM	edgeR.DESeq	edgeR.TMM.counts	edgeR.RLE	edgeR.TMM
DESeq2.DESeq	305	305	0	0	0	0	279	279
DESeq2.TMM.counts	305	308	0	0	0	0	279	280
DESeq2.RLE	0	0	0	0	0	0	0	0
DESeq2.TMM	0	0	0	0	0	0	0	0
edgeR.DESeq	0	0	0	0	0	0	0	0
edgeR.TMM.counts	0	0	0	0	0	0	0	0
edgeR.RLE	279	279	0	0	0	0	305	304
edgeR.TMM	279	280	0	0	0	0	304	307

Table 2: Number of common DE genes between all pairs of methods for the Af dataset.

4 R session information

Here are reported the versions of the packages used to perform the calculations of this document:

- R version 3.3.0 (2016-05-03), `x86_64-pc-linux-gnu`
- Locale: `LC_CTYPE=fr_FR.UTF-8`, `LC_NUMERIC=C`, `LC_TIME=fr_FR.UTF-8`, `LC_COLLATE=fr_FR.UTF-8`, `LC_MONETARY=fr_FR.UTF-8`, `LC_MESSAGES=fr_FR.UTF-8`, `LC_PAPER=fr_FR.UTF-8`, `LC_NAME=C`, `LC_ADDRESS=C`, `LC_TELEPHONE=C`, `LC_MEASUREMENT=fr_FR.UTF-8`, `LC_IDENTIFICATION=C`
- Base packages: base, datasets, graphics, grDevices, methods, parallel, stats, stats4, utils
- Other packages: Biobase 2.32.0, BiocGenerics 0.18.0, cluster 2.0.4, DESeq2 1.12.1, edgeR 3.14.0, GenomeInfoDb 1.8.1, GenomicRanges 1.24.0, IRanges 2.6.0, knitr 1.13, limma 3.28.4, RColorBrewer 1.1-2, S4Vectors 0.10.0, SummarizedExperiment 1.2.1, xtable 1.8-2
- Loaded via a namespace (and not attached): acepack 1.3-3.3, annotate 1.50.0, AnnotationDbi 1.34.1, BiocParallel 1.6.1, chron 2.3-47, codetools 0.2-14, colorspace 1.2-6, data.table 1.9.6, DBI 0.4-1, digest 0.6.9, evaluate 0.9, foreign 0.8-66, formatR 1.4, Formula 1.2-1, genefilter 1.54.1, geneplotter 1.50.0, ggplot2 2.1.0, grid 3.3.0, gridExtra 2.2.1, gtable 0.2.0, highr 0.6, Hmisc 3.17-4, lattice 0.20-33, latticeExtra 0.6-28, locfit 1.5-9.1, magrittr 1.5, Matrix 1.2-6, munsell 0.4.3, nnet 7.3-12, plyr 1.8.3, Rcpp 0.12.4.5, rpart 4.1-10, RSQLite 1.0.0, scales 0.4.0, splines 3.3.0, stringi 1.0-1, stringr 1.0.0, survival 2.39-4, tools 3.3.0, XML 3.98-1.4, XVector 0.12.0, zlibbioc 1.18.0

References

[1] Love M, Huber W, Anders S. **Moderated estimation of fold change and dispersion for RNA-Seq data with DESeq2**. *Genome Biology.* 2014; doi:10.1186/s13059-014-0550-8.

[2] Robinson M, McCarthy DJ, Smyth GK. **edgeR: a Bioconductor package for differential expression analysis of digital gene expression data**. *Bioinformatics.* 2009; doi:10.1093/bioinformatics/btp616.

[3] Zhou X, Oshlack A and Robinson MD. **miRNA-seq normalization comparisons need improvement**. *RNA.* 2013; doi:10.1261/rna.037895.112.

[4] Anders S and Huber W. **Differential expression analysis for sequence count data**. *Genome Biology.* 2010; doi:10.1186/gb-2010-11-10-r106.

[5] Robinson MD and Oshlack A. **A scaling normalization method for differential expression analysis of RNA-seq data**. *Genome Biology.* 2010; doi:10.1186/gb-2010-11-3-r25.

[6] Dillies MA, Rau A, Aubert J, et al. **A comprehensive evaluation of normalization methods for Illumina high-throughput RNA sequencing data analysis**. *Briefings in Bioinformatics.* 2013; doi:10.1093/bib/bbs046.
